# The Role of Sensorimotor Difficulties in Autism Spectrum Conditions

**DOI:** 10.3389/fneur.2016.00124

**Published:** 2016-08-10

**Authors:** Penelope Hannant, Teresa Tavassoli, Sarah Cassidy

**Affiliations:** ^1^Centre for Research in Psychology, Behaviour and Achievement, Coventry University, Coventry, UK; ^2^Seaver Autism Centre, Icahn School of Medicine at Mount Sinai, New York, NY, USA; ^3^Autism Research Centre, University of Cambridge, Cambridge, UK; ^4^Institute of Neuroscience, Newcastle University, Newcastle upon Tyne, UK

**Keywords:** autism spectrum conditions, sensory, motor, sensorimotor, repetitive behavior, cerebellum, gamma-aminobutyric acid, social cognition

## Abstract

In addition to difficulties in social communication, current diagnostic criteria for autism spectrum conditions (ASC) also incorporate sensorimotor difficulties, repetitive motor movements, and atypical reactivity to sensory input (1). This paper explores whether sensorimotor difficulties are associated with the development and maintenance of symptoms in ASC. First, studies have shown difficulties coordinating sensory input into planning and executing movement effectively in ASC. Second, studies have shown associations between sensory reactivity and motor coordination with core ASC symptoms, suggesting these areas each strongly influence the development of social and communication skills. Third, studies have begun to demonstrate that sensorimotor difficulties in ASC could account for reduced social attention early in development, with a cascading effect on later social, communicative and emotional development. These results suggest that sensorimotor difficulties not only contribute to non-social difficulties such as narrow circumscribed interests, but also to the development of social behaviors such as effectively coordinating eye contact with speech and gesture, interpreting others’ behavior, and responding appropriately. Further research is needed to explore the link between sensory and motor difficulties in ASC and their contribution to the development and maintenance of ASC.

## Introduction

Successful social functioning requires multiple skills, such as quickly seeking out and integrating information from pertinent social cues in order to plan and carry out an appropriate response. This involves effectively coordinating non-verbal and verbal language including posture, vocal-tone, facial gesture, and eye contact with speech during a social interchange. Hence, social reciprocity requires integrating a variety of sensory information from the environment to plan and execute movement effectively. If this ability was impaired, we could predict a whole host of difficulties, from performing simple actions (such as reaching for a cup) to having difficulty seeking out pertinent social cues and even difficulties interpreting others’ behavior and responding appropriately. Furthermore, novel and challenging actions may be avoided and known perfected routines preferred.

Current DSM-5 criteria (1) refers to Autism as a “disorder,” however in this paper, we use the less stigmatizing term “condition,” recognizing that Autism includes both strengths and weaknesses, while still being a medical condition for which individuals need support. Individuals with Autism Spectrum Conditions (ASC) have difficulties with social interaction and communication, repetitive behaviors, narrow circumscribed interests, and atypical sensitivity to sensory information (1). Three decades of research have attempted to uncover the cause of this diverse range of difficulties in ASC with little success. Thus some researchers have suggested that it is time to give up on finding a single unifying theory of ASC, as it may rather consist of a number of co-occurring, genetically distinct clusters of symptoms (2–4). Progress may have been hampered by lack of research into the non-social difficulties seen in ASC; repetitive behaviors, narrow circumscribed interests, and sensory difficulties. However more recent research, such as that by Gowen and Hamilton (5), has started to explore the contribution of sensorimotor difficulties (defined as an impairment in the pathway involving motor activity triggered by sensory stimuli) to the development and maintenance of ASC. This could provide a more parsimonious explanation (compared to multi-deficit accounts) of the social and non-social difficulties that come to develop in ASC. By examining both psychological and biological evidence, this paper therefore explores and hypothesises that motor coordination and sensory difficulties in ASC may be associated with the development and maintenance of ASC symptoms.

## Motor Coordination in Autism Spectrum Conditions

Unusual motor processing is associated with ASC. Initial clinical reports of ASC reported general “clumsiness” in these individuals (6–9), and this has been corroborated in more recent research (10–15). Furthermore, Fournier et al. completed a robust meta-analysis including 51 comparisons of motor ability and deduced that individuals with ASC display a pronounced motor impairment compared to neuro-typical controls, with their motor skills often fall 1.5 SDs below the typical mean (16). Green et al. (12) and Miyahara et al. (17) were able to quantify the prevalence of motor impairment by administering assessments of coordination to children with ASC, concluding that approximately 80% had definite motor impairment with 10% being borderline. The prevalence of impaired motor processing in coordination, praxis, balance, and muscle tone in ASC is also echoed in a range of other studies (5, 18–21). More narrowly, Ming, Brimacombe, and Wagner went on to identify the prevalence of specific deficits in motor skills, with hypotonia (low muscle tone) and apraxia (impaired ability to execute planned movement) being the most common deficit (51 and 34%, respectively). Additionally, a review written by Gowen and Hamilton (5) demonstrated how a number of fine and gross motor movements had been identified and reported, such as: slower repetitive hand and foot movement, slower and less accurate manual dexterity, poorer ball skills (e.g., aiming and catching), unstable balance, impaired gait (e.g., tandem gait, heel, or toe walking), reduced coordination of locomotor skills (e.g., running and jumping), and hypotonia.

Motor abnormalities in ASC are present from early infancy (22–24), such as head lag in infants (defined as the head lagging behind the trunk in a pull-to-sit position) (25). Motor delays are significantly more likely to be reported by parents as the first area of concern at a mean age of 14.7 months (26). However, Teitelbaum et al. (22) also described, in detail, coordination differences between babies with and without ASC from as young as 6 months old, such as persistent asymmetry when lying and early impairments in rolling over from back to stomach.

## Sensory Reactivity in Autism Spectrum Conditions

Atypical sensory reactivity, as defined here as the psychological reaction that occurs when a person is exposed to sensory stimuli, is also associated with ASC. Pioneering reports of ASC described sensory “intrusions” (6, 7). Studies corroborate these initial clinical descriptions, showing that the prevalence of sensory reactivity problems in ASC is high. Caminha and Lampreia (27) reported a 69–80% occurrence of sensory dysfunction symptoms in ASC, while (28) noted that 95% of their sample had some degree of sensory processing difficulties. Findings from sensory symptoms in ASC, such as hypersensitivity and hyposensitivity, vary depending on modality tested, level of analysis, and method used. Research has, for example, shown the existence of basic sensory reactivity symptoms such as: hypersensitivity across visual (29), tactile (30–32), auditory (33–36) and olfactory (37) domains as well as hyposensitivity to olfactory and gustatory stimuli (38, 39); differences in perceptual function (40); and proprioceptive impairment (ability to determine where the body is in space) (41, 42).

More narrowly with respect to visual processing, although visual acuity seems to be typical in individuals with ASC (43, 44, 45), children with ASC statistically; (a) score higher on the Embedded Figures test, thereby demonstrating greater field independence, or the ability to see objects as discrete and distinct from their background as opposed to central coherence (46, 47); (b) exhibit faster visual search times (47, 48), have enhanced discrimination ability (48); and (c) detect more unattended changes in natural stimuli when compared to typical developing children (49). Such findings of an apparent superior performance in basic sensory perception led Mottron et al. (50) to suggest that individuals with ASC have “enhanced perceptual functioning” (51). There is however evidence to demonstrate diminished performance on higher order visual processing tasks such as visual spatial and visual motion processing (52–54). Some studies have indicated that adults with ASC are impaired at motion processing but retain intact form processing (perhaps reflecting selective dysfunction of the magnocellular pathway – the motion processing pathway) (52, 54–56). Other studies have found atypical visual global processing within the dorsal visual pathway (57), or have found that attentional and cognitive demands of the tasks might contribute to differing results (58). For a comprehensive review of vision in ASC, see Ref. (53). Taken together, basic early stage visual processing seems to be similar to or superior in individuals with ASC; however, there appears to be compromised higher order visual processing, which could be linked to difficulties with temporal sensorimotor skills, such as motion processing.

Regarding auditory processing, findings are similar in nature with intact or enhanced basic auditory perception and potential difficulties with processing higher order complex sounds. For example, individuals with ASC show superior pitch processing (55, 59, 60). O’Riordan and Passetti (61) also report greater auditory discrimination ability in children with ASC, and Järvinen-Pasley et al. (62) show superior perceptual processing of speech in children with ASC. However individuals with ASC also show difficulties in filtering complex auditory sound such as speech from background noise and therefore can often have difficulty attending to or registering auditory information (63, 64).

In tactile processing results have also been mixed, showing hypersensitivity (31, 65, 66) to basic stimuli, as well as no differences (61). Another line of research shows differences in adaptation toward touch; Tommerdahl et al. (66) showed that participants with ASC outperformed controls in tactile acuity after short adaptation to a vibrotactile stimulus period of 0.5 s. In addition, they demonstrated that individuals with ASC do not show enhanced spatial localization after being adapted to tactile stimulus, which occurs in typical controls. A more recent study also showed differences in tactile processing in children and adults with ASC (67). Differences in auditory and tactile processing might be associated with sensorimotor difficulties in ASC as well as visual differences.

The chemical senses have been investigated less than other senses, showing mostly impaired or intact processing in ASC depending on measure used (38, 39). Suzuki et al. (39) for example, reported impaired odor identification using the University of Pennsylvania Smell Identification Test in adults with ASC. A second study by Bennetto et al. (38) reported that adolescents (10–18 years) with ASC were less accurate in olfactory identification. However olfactory detection thresholds seem to be intact in individuals with ASC (39, 68). Regarding taste processing, identification seems to be impaired in individuals with ASC. Bennetto et al. (38) found that adolescence with ASC are less accurate in identifying sour and bitter tastes but showed similar identification for sweet and salty tastes. In line with this, Tavassoli and Baron-Cohen (68) found that adults with ASC had difficulties in identifying bitter, sour, and sweet tastes. Moreover, on sensory questionnaires, individuals with ASC are reported to show more olfaction and taste sensitivity compared to individuals without ASC (28, 69–72). Individuals with ASC (55%) for example present with more clinical symptoms in smell/taste sensitivity on the Short Sensory Profile compared to children with Sensory Processing Disorder (32%) (73).

Kern et al. (74) reported that abnormal responsivity in each of the main sensory modalities (auditory, visual, touch, and oral) was not independent, showing significant correlations between them; suggesting that sensory responsivity dysfunction in ASC is global in nature. This is further supported by the recognition of increased rates of synesthesia in ASC (a condition in which a sensation in one sensory modality triggers a perception in another) (75). Additionally, a study by Stevenson et al. (76) recently demonstrated reduced multisensory integration [described as the process whereby information from all the different sensory modalities are combined to influence perception, decision and behavior (77)] in ASC, by using a sound-induced flash illusion as a measure. However, sensory abnormalities in ASC appear to have the potential to reduce with age: a cross-sectional, linear regression analysis with 104 participants, aged 3–56 years, suggested that sensory difficulties become similar to typical controls by the age of 33 years (78). A meta-analysis also showed that sensory symptoms seem to be most prevalent between the ages of 6–9 years of age (79).

## Impact of Motor Coordination and Sensory Reactivity in ASC

Difficulties in motor coordination and sensory reactivity have both separately been associated with the severity of symptoms in ASC. Research and primary observations indicate that the range of motor difficulties experienced by children with ASC appear to affect the development and maintenance of their social and communicative difficulties. Examples of this include the significant impairments shown by children with ASC in skilled motor gestures, including imitation (80) and development of speech sound production (20). Children with motor coordination difficulties are less competent at recognizing emotions in others (81) and are more likely to have increased anxiety on the playground due reduced social interaction (24). Furthermore, research has shown a correlation between motor and praxis performance and social communication skills in ASC (82–87). Piek and Dyck expands on the correlation and long recognized comorbidity of Developmental Coordination Disorders (DCD) and ASC, suggesting that as the disorders typically occur together, they either have overlapping causes or that one disorder is a direct cause of the other (88). However, research also suggests that impairments in movement could be a fundamental cause of the social and communicative difficulties seen in ASC, as opposed to a peripheral feature of the condition [see Ref. (89)]. This theory is consistent with recent evidence that suggests children who display fine motor difficulties early in childhood (from 7 months old) are significantly more at risk of developing an ASC by 36 months (90). Cross syndrome studies have also shown that children with ASC have more difficulties in basic (91) and gestural motor skills (92) compared to children with Attention-Deficit Hyperactivity Disorder (ADHD) and/or DCD. These results suggest that early motor difficulties are associated with the development of social and communicative difficulties later in life. Accordingly, as autism severity is based on social communication impairments in current DSM-5 criteria (1), this may be of paramount importance in the development of ASC.

In addition to the challenging sensory hypersensitivity/overload and hyposensitivity experienced by individuals with ASC, research has also linked sensory reactivity disorders to social communication difficulties (85, 93, 94). Fitzgibbon et al. (95) proposed that both physical pain and social pain are processed atypically in individuals with ASC and insensitivity to pain, for example, could in turn limit empathy and understanding of pain in others. Recent research has also identified associations between sensory reactivity and the severity of autism such as Tavassoli et al. (33) who reported that autistic traits defined by the Autism Spectrum Quotient, correlated positively with sensory over-responsivity, and Boyd et al. (96) specifically noted high levels of hyper responsive behavior predicted high levels of repetitive behavior. Other such studies include: Ashwin et al. (37), Kern et al. (74), Hilton et al. (85), Ben-Sasson et al. (79), Lane et al. (97), Siaperas et al. (98), and Tavassoli et al. (99).

## Sensorimotor Integration in ASC

The evidence reviewed so far show that sensory and motor difficulties are prevalent in ASC and impact on social functioning (86, 93). Evidence also suggests that these difficulties are present from birth, e.g., pre-social skill deficits (29, 100), and increase the risk of developing ASC by aged 3 years (90). This indicates that there is a possible impairment in the process of sensorimotor integration [a brain process that allows, by complex neural operations, the connection of the sensory and motor domains (101), p. 427] that plays a fundamental role in the development of ASC. Although further studies are needed to explore whether sensorimotor integration difficulties are unique to ASC, Gowen and Hamilton (5) also proposed that altered sensory input and variability in motor execution “together” may play a pivotal role in ASC. Researchers have linked weaker praxis and motor performance to sensory reactivity in ASC (98, 102–104). Additionally, Siaperas et al. (98) found that children with ASC demonstrated significant impairment in both motor performance and proprioceptive and vestibular processing and thus suggested that sensory difficulties are not a peripheral, but a core feature of ASC.

Sensory feedback and movement are intrinsically connected (105), as the ability to plan and execute a simple movement effectively (such as reaching for a cup), requires sensory feedback (such as your position in relation to the cup as you reach for it) in order to effectively coordinate movement while performing the action (5, 105). Any error signal (such as missing the cup) at the final stage of movement is then processed and corrected. As movements are repeated in this fashion, they become automatic, and the delay caused by continuous sensory feedback is reduced, as the motor command (feedforward program) rapidly generates a prediction of the sensory consequences of the action (106, 107). For this reason, when sensory guidance is unreliable, slow, or associated with negative effect, both the ability to first acquire a motor command, in addition to regulating a stored motor command, would be impaired, leading to limited accuracy and flexibility (105). Therefore, deficiencies in sensorimotor integration would present as difficulties in effectively utilizing sensory feedback to correct movements, resulting in coordination difficulties and sensory reactivity abnormalities comparable to those seen in ASC (100, 105–112).

A number of studies have shown difficulties in sensorimotor integration in ASC. For example, Ronconi et al. (113) demonstrated that visual attention was impaired in children due to an imbalance of sensorimotor feedforward and feedback programs, by demonstrating a slower zoom-in and zoom-out mechanism in the eye. Schmitt et al. (114) and Mosconi et al. (115) demonstrated that those with ASC were significantly less accurate when moving their eyes from a central fixation to a peripheral target, showing increased saccade variability and difficulties in decelerating saccades. Wilkes et al. (116) also showed that children with ASC were delayed in initiating a saccade when following a moving light with their eyes compared to controls. Price et al. (117) demonstrated compromised visual sensitivity to human motion, and Glazebrook et al. (118) showed that adults with ASC had difficulty coordinating both hand and eye movements, taking significantly longer to complete integrated tasks than typical controls. These low level difficulties in initiating and adjusting saccades, and coordinating hand and eye movements, could explain a range of social and communication difficulties seen in ASC. For example, delay in looking to pertinent social cues (119–121), particularly for fast paced dynamic stimuli (122–125), with resulting difficulties in early social engagement and later ability to interpret others emotions and behavior (122, 123, 126, 127).

Studies have also shown that those with ASC have difficulty integrating sensory information in motor learning. For example, when children with ASC performed a motor learning task on a touch screen, the presence of a visual distractor did not impact their performance like with typical controls (109). Gepner suggested a correlation between visuo-postural detuning and ASC severity, whereby individuals with ASC had weaker postural stability and reactivity to environmental motion (128). Similarly, children with ASC are significantly less able to correct movements from visual compared to proprioceptive feedback (128–131). Studies have also shown difficulties specifically with motor movements, which require integrating visual cues or other sensory signals (18, 103, 132), and children with ASC have difficulty specifically when tracing shapes using feedback from a mirror image, and imitating others actions (133). These results all suggest that those with ASC do not tend to incorporate other sensory inputs, particularly visual feedback, into motor learning and have difficulty coordinating visual and motor movements. These difficulties could particularly impact social learning from imitation and integration of eye movements with gesture during social communication in ASC. However, further research is needed to explore this possibility.

Despite the possible interpretations of perceptual feedback being incorrect, Vandenbroucke et al. (134), by using a forced-choice texture segregation task, went on to suggest that with considerable practice individuals with ASC were able to compensate for the imbalance in feedback and build on a feedforward program. Larson et al. (135) also noted that the mechanisms of acquisition and adaptation of feedforward programs are indistinguishable between children with ASC and typically developing children. Furthermore, Gowen et al. (132) demonstrated that in comparison to neurotypical controls, individuals with ASC rely to a greater extent on a goal directed pathway, established in part by a feedforward program. Similarly, Rinehart et al. (136) demonstrated an intact ability to execute programed movement but atypical movement preparation. Nazarali et al. (137) demonstrated that individuals with ASC have difficulty reprograming already planned movements when given additional sensory information, and Glazebrook et al. (118) showed that individuals with ASC can use sensory input such as vision and proprioception, although the greater visual-proprioceptive integration required the more time was taken by the ASC group to perform the movements. Thus, with age, through continual practice, coping strategies and natural development, known, repetitive movements and feedforward motor programs appear to improve in ASC.

## Biological Basis of Impaired Sensorimotor Integration in ASC

In addition to psychological research given above linking sensorimotor integration to ASC, there is a substantial amount of biological evidence that collaborates this relationship. For example, difficulties in sensorimotor integration in ASC have been linked to the cerebellum, such as saccadic accuracy being connected to impairment of the error-reducing function of the cerebellum (114). Other examples include a correlation in the magnitude of cerebellar hypoplasia with decreased exploration in children with ASC (138), and an association of cerebellum volume with specific difficulties incorporating visual cues in motor learning (129). The cerebellum is reported to contain pathways that link sensory signals to motor areas in the brain (139), and these are important in controlling and coordinating movement (140). Stoodley and Schmahmann (141) specifically showed an anterior sensori-motor versus posterior cognitive/emotional dichotomy in the cerebellum. Research has also shown that the cerebellum has a fundamental role in maintaining the equilibrium between feedback and feedforward programs in sensorimotor integration, for example, Kawato et al. (142) found that the cerebellum was the most likely site for feedforward programs to be stored, by using functional magnetic resonance imaging (fMRI) to measure specific brain activity during coordinated and planned movement; Brooks (105) and Mostofsky et al. (143) noted that the cerebellum was responsible for triggering learned movement (feedforward programs); and Fuentes and Bastian (144) suggested that the cerebellum is intrinsic to predicting movement outcomes.

Abnormalities in the cerebellum of individuals with ASC are one of the most consistent neuroanatomical findings (145). McAlonan et al. (146) found structural abnormalities in the cerebellum with deficits in gray and subcortical white matter. Using fMRI, atypical patterns of both cerebral activation (indirectly detected by increased cerebral blood flow) and deactivation (signaled by decreased cerebral blood flow) have been noted in ASC: where cerebral activations during a simple motor movement were found to be mainly confined to the anterior cerebellum in TD adults but also spread to the posterior cerebellum in ASC adults (147–149). Additionally, 95% of autistic cerebella examined at autopsy showed clearly defined anatomic abnormalities; most commonly a significantly decreased number of Purkinje cells, a large inhibitory neuron thought to regulate motor function (145, 150, 151).

Moreover, the basal ganglia, which is considered to be reciprocally connected to the cerebellum (152), is also reputed to play a functional role in both motor and sensory control and integration (152, 153). More specifically, it is hypothesized that within the basal ganglia, there are two distinct striatal pathways that facilitate both movement and sensory representation. Although it is unclear whether these are distinct or seemingly intertwined (154), a direct pathway appears to be responsible for facilitating movement whereas an indirect pathway is thought to inhibit both competing motor programs and afford sensory control by filtering and gating sensory input (155). The basal ganglia have been shown to have decreased volume in ASC (156). Furthermore, the striatum, one of the largest components of the basal ganglia, is reported as having excess functional connectivity in ASC (157).

In addition to anatomical differences of the cerebellum and basal ganglia being associated with sensorimotor impairment in ASC, both brain regions contain large GABAergic inhibitory neurones. More specifically, Purkinje cells, considered the sole output of all motor coordination in the cerebellar cortex (158) and “medium spiny neurons,” thought to form 95% of the striatum in the basal ganglia (159). The inhibitory neurotransmitter GABA (gamma-aminobutyric acid) and the main excitatory neurotransmitter glutamate released by these neurones also play an important role in sensory discrimination in ASC (160). GABA is known to decrease the firing of neurons (161), thereby reducing and inhibiting sensory feedback. Alterations in GABAergic transmission have been associated with sleep disorders (melatonin production) (162), mood disorders, anxiety and other hyper-excitable states such as epilepsy (163, 164). GABA levels have also been shown to be lower in the auditory and motor cortices of children with ASC with a mean deficiency of GABA equating to 22 and 11%, respectively, in comparison to TD peers (165) GABAergic functioning has been implicated in tactile reactivity (166, 167). Moreover, reductions in GABAergic system have been discovered in ASC brain tissue: with significant reductions in GABA_A_ receptors, 63% reduction in comparison to controls (168), and a reduction by 61% of the glutamic acid decarboxylase protein (the enzyme responsible for converting glutamate into GABA) (169). Similarly, increased glutamate levels (excitatory neurotransmitter) in blood and platelets have been found in ASC subjects, suggesting impaired conversion of glutamate to GABA, consequently increasing the excitatory state of the brain (169, 170). A GABA receptor gene, *GABRB3*, is one of the key candidate genes for ASC as found in humans as well as in animal models (171, 172). A study by Green et al. (173) demonstrated that participants with ASC also showed stronger activation of the amygdala toward sensory stimuli, which is thought to perform a pivotal role in emotion processing and decision-making; GABAergic neurons are also present in the amygdala.

Further evidence of an imbalance in these vital neurotransmitters in ASC arise from treatments for hyperactive disorders of the auditory system, such as tinnitus and hyperacusis (a lowered threshold for discomfort from sounds that typical individuals do not find unpleasant) (174), where the administration of benzodiazepines such as Clonazepam (an allosteric modulator of the GABA_A_ receptor) have been used to restore the balance between inhibition and excitation in the brain (174). Banji et al. (175) also demonstrated that induced cerebellar damage in mice instigated motor clumsiness, similar to that seen in ASC, the motor clumsiness was then reduced by treating the mice with green tea extract (*Camellia sinensis*). l-Theanine is a major amino acid component found almost exclusively in green tea (176–178) and blocks the binding of l-glutamic acid to glutamate receptors in the brain (176, 178, 179), thereby perhaps aiding the improvement in motor activity by increasing inhibition of movement.

## Impact of Sensorimotor Difficulties in ASC

Further evidence to support our hypothesis can be found in studies that have demonstrated sensorimotor difficulties and associated biological markers specific to ASC. In particular, difficulties with accuracy, speed, and initiation of eye movements; coordination of eye and body movements; and the ability to integrate visual information into motor learning could all have a profound impact on social learning opportunities during development and maintenance of social and communication difficulties in ASC. For example, difficulty quickly moving and correcting saccades could explain the well-established lack of attention to social cues in young children who go onto develop ASC (180, 181), with a cascading effect on later social development and learning (124). Difficulties integrating eye movements with body movements could account for social communication and interaction difficulties in ASC such as integrating eye contact with gesture and speech (1). Difficulties integrating other cues, particularly visual information in motor learning, could explain the challenges faced in social imitation in ASC [e.g., Ref. (182)]. Social imitation is key for social learning and could also contribute to the development and maintenance of social difficulties in ASC. Sensorimotor impairment could also explain other autistic traits such as echolalia and repetitive behaviors. A major study in brain anatomy using MRI by McAlonan et al. put forward that the impaired inhibition of sensory feedback through defective sensory gating (the brain’s selective processing of sensory stimuli) found in ASC, could lead to difficulties where the individual is unable to inhibit repetitive thoughts, actions, or speech (146). Indeed, non-ASC related research has already demonstrated links between sensorimotor control and social behavior. Skewes et al. (183), by noting how the size and precision of a visual illusion may influence visual motor behavior, suggest that potentially, the way in which sensorimotor control adapts to the opinions of others may help facilitate smoother social interaction. Hoke et al. (184) identified that the integration of sensory and motor processing underlies social behavior in tungara frogs.

A number of theories have attempted to explain the development of ASC, including ability to understand mental states to predict others behavior (Theory of Mind) (185); impaired eye gaze detection (186) or lack of early social attention in favor of objects (124). These theories have failed to explain the wide range of difficulties seen in ASC from social communication to sensory reactivity and repetitive motor movements. Sensory and motor difficulties have also been considered largely peripheral to ASC, with atypical sensory reactivity only recently being added to DSM-5 diagnostic criteria (1). However, the wide range of difficulties in ASC could be explained by using a wider perspective: a central theory of sensorimotor integration impairment and the ensuing “chain” of likely misalignments and misjudgments that follow. Early development theories such as Jean Piaget’s developmental stage theory proposed that sensorimotor integration was central to neurotypical development and where a child struggles to coordinate their initial sensory experiences, further stages of development will be impaired (187).

## Future Directions

To the authors’ knowledge, there are no studies that have explored the impact of sensorimotor difficulties (such as saccades or impaired motor learning) on the development or maintenance of core ASC symptoms. Sensory feedback and feedforward programs are seemingly pivotal to successful sensorimotor integration. A greater understanding of these systems and the impact of sensorimotor integration in ASC may be a crucial way forward to understanding the development and maintenance of this condition. For example, research that identifies a significant correlation between motor coordination, sensory reactivity, and the severity of ASC could demonstrate that these are fundamental and pervasive difficulties associated with and reflective of the scale of the condition. Similarly, further research into the varying degrees of sensorimotor difficulties and more specifically at the level the difficulties occur, such as the more complex higher order level including anticipation and timing, may also be crucial in identifying if and which area the sensorimotor chain is affected in ASC. There also appears to be a critical window for the impact of such sensorimotor deficits on cognitive and social development: below the age of two. Consequently, interventions incorporating both structured physical tasks and sensory environments below this age should have particular focus. Such intervention is also recommended in Barenek’s review of the efficacy of sensory and motor interventions in ASC (2002), where it is noted converging evidence would suggest beginning sensorimotor inventions at an early age may be beneficial. Similarly, sensorimotor integration difficulties in comorbid conditions, such as dyslexia and dyscalculia should also be explored, as finding a recurrent thread to specific learning difficulties in ASC could alter the type and time of intervention.

Additionally, the apparent deficiency of the inhibitory neurotransmitter GABA in the cerebellum of ASC individuals should also be an area for consideration, as this could have a global impact on sensorimotor planning, cognitive and social development. The introduction of a non-evasive GABA substitute, such as oolong tea, could therefore lead to a decrease in sensory feedback, supporting an equilibrium with feedforward programing and ultimately moderate planned movement.

## Conclusion

To conclude, we hypothesize that social communication, interaction difficulties, and repetitive behaviors in ASC appear to be associated with motor coordination and sensory reactivity, specifically attaining and coordinating the delicate balance between the feedforward programs and feedback systems of sensorimotor integration. However, once reached, research indicates that in comparison to controls, the feedforward program can be maintained and utilized just as efficiently in ASC provided environmental cues stay the same. Such conclusions are reinforced when listening to the lived experiences of ASC individuals; “I can do buttons up fine, unless I concentrate too hard.”

All studies included in this research have been summarized in Table S1 in Supplementary Material.

## Author Contributions

PH: developed topic, literature search, compiled evidence, wrote the manuscript, and completed the table. SC: literature search, compiled evidence, critical feedback, wrote the manuscript, and completed the table. TT: literature search, compiled evidence, critical feedback, and wrote the manuscript. All authors read and approved the final manuscript.

## Conflict of Interest Statement

The authors declare that the research was conducted in the absence of any commercial or financial relationships that could be construed as a potential conflict of interest.

## Supplementary Material

Table S1 **Summary of all studies included in the role of sensorimotor difficulties in the development of autistic spectrum conditions**. Summary of demographics, methods and outcomes of all studies included in research.

## Abbreviations

ASC, autistic spectrum conditions; GABA, gamma-aminobutyric acid.
