# Supplementary material for: The Role of Sensorimotor Difficulties in Autism Spectrum Conditions
Source: Front Neurol. 2016 Aug 10;7:124. doi: 10.3389/fneur.2016.00124 (PMC4978940; doi:10.3389/fneur.2016.00124)
Supplement: Table S1 — Summary of all studies included in the role of sensorimotor difficulties in the development of autistic spectrum conditions. Summary of demographics, methods and outcomes of all studies included in research. [file Table_1.DOCX]

Summary of All Studies Included in A Review of Sensorimotor Difficulties in the Development of Autistic Spectrum Conditions)]

| **Study Source** | **Participant Demographics** | **Study Type(s)** | | | **Methods / Tasks Utilised** | **Findings / Outcome of Study** |
| --- | --- | --- | --- | --- | --- | --- |
|  |  | **Sensory** | **Motor** | **Sensori-**  **motor** |  |  |
| **Motor Coordination - Impairment in Anticipation** | | | | | | |
| *Brisson et al. (2012)* | 13 infants (aged 4-6 months) later diagnosed with ASC and 14 typical controls. |  | YES |  | Retrospective analysis of home videos of feeding situations; whether the infant opens their mouth in anticipation of the spoon’s approach. | Infants with ASC showed significantly less anticipatory mouth opening in response to the approaching spoon. This suggests early motor anticipation difficulties in ASC. |
| *Cattaneo et al. (2007)* | 8 High functioning ASC children and 8 typical controls, right handed, aged 5-9 years old, FSIQ>70, diagnosis confirmed with ADI-R and ADOS in clinic. |  | YES |  | Mouth opening muscle recorded while a) observing and b) carrying out two actions; grasping food with the right hand, bringing to the mouth and eating it; grasping a piece of paper and putting it in a container on the right shoulder. Ankle muscle activation recorded while participants grasped food with the right hand, and placing it into a container that had to be opened by pressing a foot pedal; and grasping a paper ball and placing it in an open container, keeping their foot still on the pedal. | Children with ASC showed significantly reduced activation of the mouth opening muscle when a) observing and b) carrying out actions involving reaching for food, unlike typical controls. Children with ASC only showed mouth muscle activation when bringing food to the mouth. |
| *Rinehart et al. (2001)* | 11 individuals with High Functioning Autism (10 male), 12 controls matched on age, FSIQ and gender, and 12 individuals with Asperger Syndrome (10 male), aged 6-19 years. IQ>70. ASC diagnosis confirmed by ADI-R. |  | YES |  | *Motor reprogramming task;* participants rapidly press left and right target buttons (cued by flashing light). An ‘Oddball’ was interspersed in these trials requiring the participant to quickly alter their response to an outlying left or right target. | Individuals with autism and Asperger syndrome have atypical movement preparation with an intact ability to execute movement. The atypical deficit in motor preparation found in autism was characterised by a ‘lack of anticipation’ in autism. |
| **Motor Coordination - Difficulties in ASC** | | | | | | |
| *Glazebrook et al. (2009)* | 13 high functioning young adults with ASC (11 male), and 15 typical controls (13 male), mean age 23 years. |  | YES |  | Participants performed eye movements and/or manual reaching movements when cued towards a left/right target. In one condition, visual feedback when reaching towards the target was not available. | Young adults with ASC used advance information to plan their movements (hand or eye separately). However, they experienced difficulties in coordinating their eye and hand movements. |
| *Jansiewicz et al. (2006)* | 40 male children and adolescents with ASC, and 55 typical control males, aged 6-17 years, FSIQ>80. ASC diagnosis confirmed with ADOS and ADI-R. |  | YES |  | Physical and Neurological Exam for Subtle Signs (PANESS). | The ASC group was shown to have significant difficulties on several measures of motor control compared to the typical control group. These motor signs (PANESS variables) distinguished boys with high-functioning ASC from typical controls. |
| *Kopp et al. (2010)* | 131 females with ASC and/or ADHD, and 57 female typical controls, aged 3 – 18 years old, without learning disability. |  | YES |  | M-ABC, EB-Test (physiotherapy protocol to measure a range of motor skills), WISC-III, Autism Spectrum Screening Questionnaire, Conners’ Teacher Rating Scale-Revised (for ADHD symptoms), Vineland Adaptive Behaviour Scale - daily living skills domain, Global Assessment of Functioning Scale, Parent Questionnaire (for ADHD and comorbidities), Medical motor examination. | 25% of school girls with ASC, 32% with ADHD, and 80% preschool girls with ASC, meet diagnostic criteria for DCD. Their motor problems contribute to reduced activity in daily life even when the effects of performance IQ have been controlled for. |
| *MacNeil & Mostofsky (2012)* | 24 children with ASC,  24 children with ADHD, and 24 typical control children, aged 8-13 years. ASC diagnosis confirmed with ADI-R and ADOS. IQ above average. |  | YES |  | Physical and Neurological Exam for Subtle Signs (PANESS), praxis measure (skilled gestures on command or imitation, and tool use), and the Postural Knowledge Test (PKT). | Children with ADHD and ASC showed significant difficulties in basic motor control. However, performance and recognition of skilled motor gestures consistent with dyspraxia were shown to be specific to ASC. |
| *Milne et al. (2006)* | 23 children with ASC (22 males), and 23 typical control children (10 males), aged 8-12 years. Non-verbal IQ 70-130. |  | YES |  | 2D:4D digit ratio (indicator of fetal testosterone), coherent motion detection task, coherent form detection task, motor control tasks (bead threading, balance, finger/thumb rotation, and heel-to-toe walking). | Children with ASC had significantly lower 2D:4D digit ratios than the control group, suggesting that foetal testosterone levels are higher, as this ratio is more prominent in males. The ASC group also showed impairment of both fine and gross motor control. |
| *Page and Bouchert (1998)* | 33 children with ASC (25 male), aged 5-16 years old, 10 with co-morbid conditions (Fragile X, epilepsy and Trisomy 13 and 15). |  | YES |  | *Oromotor function*; manipulating direction of tongue out, side of mouth, to chin and nose. Lip movements around a whistle and blowing, mouth closure during eating and presence of dribbling.  *Hand skills;* producing a sign with two hands (bimanual coordination), forming a sequence of hand shapes, handling of everyday objects (e.g. toothbrush and hairbrush).  *Gross motor function;* informal observation of running, jumping, hopping, posture, and gait. | 70% of children with ASC had oromotor impairments; 55% had manual impairments; and 17% had gross motor impairments. |
| *Staples & Reid (2009)* | 25 children with ASC (21 male), aged 9-12 years, FSIQ 34 – 104, and 3 typically developing comparison groups matched on: (a) sex and chronological age (21 male, 4 female, aged 9-12 years), (b) movement skill performance (18 male, 4 female, aged 4-6 years), and (c) mental age (16 male, 3 female, aged 4-10 years).  ASC diagnosis confirmed by ADOS, and SRS. |  | YES |  | Test of gross motor movement (TGMD-2), Autism Diagnostic Observation Schedule (ADOS) Social Responsiveness Scale (SRS), and Leiter-R (measure of non-verbal ability). | Children with ASC performed significantly lower on the TGMD-2 than their age matched peers, and those matched on mental age. Children with ASC performed similarly to children half their chronological age. This suggests both delayed and atypical development of motor skills in children with ASC, resulting in a deficit in these skills. |
| *Whyatt & Craig (2012)* | 18 children with ASC (11 male), aged 7 to 10 years, and two age matched control groups; a) receptive language matched (6 males, 13 females, aged 8-14 years); and b) non-verbal IQ matched (11 males, 11  Females, aged 8-10 years). |  | YES |  | Movement ABC (M-ABC), British Picture Vocabulary Scales II (BPVS-II) and Wechsler Nonverbal Scale of Ability (WNV). | Evidence of general motor impairment in the ASC group compared to both age matched control groups. Universal significant specific deficits in ASC found in only 2/8 subcomponent skills; catching a ball and static balance. Motor skill deficits in ASC may be more apparent in activities demanding complex, interceptive actions or core balance ability. |
| **Motor Coordination - Correlation Between Social Communication** | | | | | | |
| *Cummins et al. (2005)* | 39 children (17 female, 22 male) with Motor Difficulties (MD) aged 6y 11m- 12y 11m, VIQ 83-145, PIQ79-146  39 typical control children (17 female, 22 male) aged 6y 11m, 12y 11m, VIQ 83-151, PIQ 87-155. |  | YES |  | Heirarchical regression used to explore the relationship between social problems (internalizing and externalizing behaviour problems; CBLC) and fine and gross motor skills (MAND), while controlling for emotion recognition ability (ERS) and IQ (WISC-III). | Children with motor coordination problems are less accurate and slower in responding to facial emotion cues.  The level of motor ability in children with MD was negatively correlated with social problems. |
| *Dziuk et al. (2007)* | 47 children with ASC (43 males), 47 typical controls (41 males), aged 8-14 years, FSIQ>80. ASC diagnosis confirmed with ADOS and ADI-R. |  | YES |  | Praxis examined using the Florida Apraxia Screening Test for children (REF). Basic motor skills assessed using the Physical and Neurological Assessment of Subtle Signs (PANESS, REF). | The ASC group continued to show poor praxis compared to the TD group, even when accounting for basic motor skills. Praxis performance and social communication skills (ADOS) were correlated in ASC, even when accounting for basic motor skill performance. |
| *Ghaziuddin and Butler (1998)* | 12 children with Aspergers Syndrome (11 males, 1 female), mean age 11.4y, mean FSIQ 104.9  12 children with Autism (11 males, 1 female), mean age 10.3y, mean FSIQ 78.4  12 children with PDD-NOS (10 males, 2 females), mean age 10.1y, mean FSIQ>78.2  Diagnosis confirmed with the Autism Behaviour Checklist |  | YES |  | Standardised (Bruinink Oseretsky) test of motor coordination administered to all three groups by a trained investigator who was blind to group membership. | All children with ASC had difficulties with motor coordination. Moreover, those with autism and PDD-NOS had more severe difficulties than those with Asperger syndrome, only when IQ was not taken into account. Suggests differences in IQ may explain differences in motor coordination ability between ASC subgroups. |
| *Hilton et al. (2007)* | 51 children with Asperger syndrome (44 males), 56 typical control children (45 male), aged 6-12 years. |  | YES |  | Movement ABC (M-ABC), and the Social Responsiveness Scale. | 89% of AS group scored 1 SD below the norm, and 65% below the 5^th^ percentile on the M-ABC. Strong correlations were found between the M-ABC motor impairment levels and the SRS severity levels. Indicating that motor skill impairment is a function of symptom severity within AS. |
| *MacDonald, Lord & Ulrich. (2013)* | 194 children with ASC, and 39 typical control children, aged 14-49 months. ASC diagnosis confirmed with ADOS. |  | YES |  | Mullen Scales of Early Learning (providing motor skills measures), and Vineland Adaptive Behaviour Scales. | Fine and gross motor skills significantly predicted participants’ adaptive behaviour skills. |
| **Motor Coordination - Programme Chaining Difficulties** | | | | | | |
| *Fabbri-Destro, Cattaneo, Boria & Rizzolatti (2009)* | 12 children with ASC (11 males), and 14 control children (8 males), aged 5-12 years, IQ>70. ASC diagnosis was made with ADOS. |  | YES |  | Children placed an object into a small or large container with the right hand. | In children with ASC the kinematics of the first motor act was not modulated by task difficulty. Suggests children with ASC have difficulty chaining motor acts into a global action. |
| *Nazarali, Glazebrook & Elliott (2009)* | 12 male participants with ASC, 12 typical controls (10 male), aged 19-34 years. |  | YES |  | Participants received a pre-cue (left or right hand) prior to a receiving a specifying target location (e.g. left hand – left target). In another experiment, participants received pre-cues consistent (80% of trials) or inconsistent (20% of trials) with target location. | Individuals with ASC have significantly greater difficulty reprogramming already planned movements than typical controls, particularly when a different hand (as opposed to direction) is required. |
| **Sensory Processing – Olfactory and Taste** | | | | | | |
| *Ashwin et al. (2014)* | 17 adult males with ASC (mean age 37.9 years, mean IQ 123.5), and 17 controls (mean age 27.2 year, mean IQ 122.7) matched on gender and IQ. | YES |  |  | Autism Spectrum Quotient (AQ), and the Alcohol Sniff Test (AST), a standardised measure of olfactory thresholds. | The ASC group showed significantly greater olfactory sensitivity than controls, detecting the alcohol odour at a mean distance of 24.1 cm from the nose, compared to 14.4 cm for controls. Mean distance olfactory detection scores were significantly correlated with self-reported autistic traits in the ASC group. |
| *Bennetto, Kuschner and Hyman (2007)* | 21 participants (17 male, 4 female) with Autism, aged 10-18 years (mean age 14.35y, FSIQ: 105.62  27 typically developing control participants (20 male, 7 female) (mean age: 14.48y, FSIQ: 109.73  ASC diagnosis confirmed by the ADI-R and ADOS | YES |  |  | A test of taste identification using sucrose, NaCl, Citric Acid and Quinine solutions. The solutions were applied to the standard locations on the anterior tongue. Electrogustometry used to establish taste detection thresholds. Olfactory identification was assessed with the ‘Sniffin’ Sticks’ Odor Identification Screening Test. | Children and Adolescents with Autism showed significantly lower accuracy in comparison with control group. Participants in both groups did not differ in accuracy when identifying sucrose or salt, but had difficulty in identifying citric acid or quinine. Results suggest that the Autism group have difficulty in identifying ‘sweet’ tastes. |
| *Suzuki et al. (2014)* | 12 males with Asperger’s syndrome, with a mean age of 33years and with a mean estimate FSIQ of 107.  12 male matched controls, mean age of 31 years, with a mean estimate FSIQ: 112.  Diagnosis confirmed by meeting both ICD-10R criteria for Asperger’s syndrome and ADI criteria. | YES |  |  | Participants were required to detect odors, starting at a strong concentration of odorant (4%) and decreasing. Participants were given two forced choices, blank or odorant. Olfactory identification ability was also assessed using UPSIT, utilising a scratch and sniff test of 40 odorants, with a forced choice of 4 alternatives per item. | There was no significant difference between the AS and control groups in the first odor detection task. However, the AS group showed significantly less accuracy when it came to the Olfactory identification task, making more errors than the control group. |
| *Tavassoli and Baron-Cohen (2012)* | Experiment 1:  38 ASC participants, mean age 35:9 and a mean FSIQ 112.6.  Ratio of 18 female: 20 male.  42 neurotypical participants, mean age 28:8 and a mean FSIQ 115.7.  Ratio of 20 female: 22 male.  Experiment 2:  19 ASC participants, mean age 28 and a mean FSIQ 113.  Ratio of 8 female: 11 male.  19 neurotypical participants, mean age 30 and a mean FSIQ 118.  Ratio of 8 female: 11 male.  ASC diagnosed by meeting DSM-IV criteria. | YES |  |  | Experiment 1:  Olfactory Detection was measured using the ‘Sniffin’ Sticks Olfaction Test’ (Burghart, Messtechnik, Germany).  Experiment 2:  Olfactory adaptation was measured using a repeated and prolonged exposure to butanol and the detection threshold was otherwise measured as for Experiment 1. | This study demonstrated that both adults with and without ASC did not differ overall in olfactory detection thresholds. Furthermore, adults with ASC showed normal adaptation to an olfactory stimulus, i.e. being less sensitive to an olfactory stimulus after prolonged exposure. |
| **Sensory Processing - Tactile** | | | | | | |
| *Blakemore et al. (2006)* | 16 participants with ASC (3 females, 13 males), mean age 27.3, FSIQ 71-138  16 neurotypical controls (9 females, 7 males), mean age 33.9, FSIQ not given for all NT participants.  ASC diagnosed independently by clinician | YES |  |  | Tickling equipment used to produce the tactile stimulus consisted of a piece of foam attached to a plastic rod which was mechanically limited to vertical sinusoidal movements of amplitude 1.5cm | The AS group showed normal attenuation of self-generated touch, however the results also demonstrated hypersensitivity in AS for both external and self-generated stimulation. The AS group rated the tactile stimuli as significantly more tickly than did the control group. |
| *Cascio et al. (2008)* | 8 adults with HFA (7 male, 1 female), mean age 28.3 with a FSIQ of at least 70.  8 control participants (7 male, 1 female), mean age 29.0.  ASC diagnosis confirmed using ADI-R and ADOS. | YES |  |  | Participants were required to complete 2 separate, identical sessions, each consisting of 5 paradigms (Contact Detection Thresholds, Vibrotactile Detection and Adaption, Hedonic Magnitude Estimation of Textured Surfaces, Thermal Sensation Thresholds and Thermal Pain Thresholds) to measure the different aspects of somatosensory perception. During each paradigm measurements were taken from both the hairy skin from the right dorsal forearm and the glabrous skin of the right thenar palm. | he thresholds on several aspects of tactile sensitivity were similar between individuals with autism and controls. This suggests that the differences in tactile sensitivity in autism is not due to a difference in detecting light pressure on the skin. |
| *Puts et al., (2014)* | 32 children with ASC (5 females, 27 males) mean age 10.7 years, mean FSIQ 103.14.  67 typically developing children (13 females, 54 males) mean age 10.08 years, mean FSIQ 117.33  All ASC children met DSM-IV criteria for ASC which was then confirmed by ADOS-G and ADI-R | YES |  |  | A cortical metrics tactile simulator was used to deliver varying stimuli to the skin on digits 2 and 3 of the left hand. The vibrotactile tasks completed included: reaction time; static and dynamic detection threshold; amplitude discrimination; frequency discrimination; and temporal order judgment. | The results indicated that there are significant differences in tactile sensitivity between children with and without ASC and also suggested a possible impairment in the somatosensory inhibitory system in ASC. |
| *Tommerdahl et al. (2007)* | 4 males with Autism, age range 21-42 years and FSIQ range from 87-129.  Control participants unknown.  Diagnosis confirmed using the ADI-R and ADOS. | YES |  |  | Participants were required to complete a tracking experiment, consisting of 2 forced choices. Each trial consisted of three stimuli (1) an adapting stimulus (either 5 or 0.5 s in duration); (2) a standard stimulus (0.5 s) delivered at the same site as the adapting stimulus, and (3) a test stimulus (0.5 s). | Participants with autism outperformed the control group on the tracking task, however the subjects with autism did not improve on tactile localization performance even when the duration of adapting stimulation was lengthened. |
| **Sensory Processing - Visual** | | | | | | |
| *Bertone, Mottron, Jelenic and Faubert (2005)* | 13 HFA individuals, mean age 22y 3m, mean FSIQ 100.4.  13 typically developing control participants, mean age 20y 5m, mean FSIQ 108.2.  ASC diagnosis confirmed using ADI and ADOS-G. | YES |  |  | Orientation Identification Task- In a dim lit room, participants were required to identify the orientation (vertical or horizontal) of the stimuli, presented for 750ms. Before testing began, participants were given practice trials to familiarize themselves with fixation, stimuli presentation and responding.  Flicker Sensitivity Task: Participants were presented with trials of flickering stimuli, and asked to identify the trial with the stimuli, either during first or second presentation. | Orientation Task- Findings show that the HFA group outperformed the TD control, suggesting superior ability in physical stimuli detection and visuo-spatial information processing.  Flicker Task- Participants from both HFA and TD groups did not differ in performance. These findings suggest that HFA participants demonstrate intact simple motion processing. |
| *Jolliffe and Baron-Cohen (1997)* | 17 HFA participants with a mean age of 30.71years and a mean FSIQ score of 104.59.  17 Asperger’s syndrome participants with a mean age of 27.77years and a mean FSIQ score of 107.06.  17 normal adult controls with a mean age of 30 years and a mean FSIQ score of 106.18.  A ratio of male to female was 15:2 across all 3 groups.  Diagnosis confirmed in the AS and HFA groups by DSM-IV and ICD-10 criteria. | YES |  |  | Experiment 1: Participants were required to complete Forma A of the Embedded Figures 12 card Test, which required participants to find the embedded simple shape within a complex design on the card. They were given a time limit to trace the embedded figure.  Experiment 2: With the same participants, the objective was to draw a pattern from memory after being given 1 minute to look at it. Subjects were scored on a) number of lines used to complete the pattern and b) whether the global outline was drawn first or later. | Experiment 1: The findings for Experiment 1 show that HFA and AS participants outperformed their control group on the EFT, suggesting superior performance and a higher intelligence level.  Experiment 2: Neither HFA or AS groups differed from the control group on the task. However, findings show that the Autism groups were more likely to draw the global lines later than the control group did. Furthermore, the groups with autism were more likely to draw more lines and draw in a more fragmented manner. Findings suggest that the autism groups show a fragmented way of remembering (e.g. like a jigsaw), whereas the control group were more likely to remember the opposite way. |
| *Koldewyn, Whitney and Rivera (2011)* | 16 ASD adolescents (14 male, 2 female), mean age of 15.4 and a mean FSIQ score of 110.6.  16 typically developing adolescents (14 male, 2 female), mean age 15.6 and a mean FSIQ score of 118.6.  The ASD group’s diagnosis was confirmed using the ADOS. | YES |  |  | Coherent Motion stimuli: Participants were assessed using the Global Dot Motion task. Each dot was given a direction (left or right). Participants had to indicate the direction of the global motion.  Biological Motion stimuli: Introduced noise into a visual motion display of a walking body. The walker remained consistent throughout the task, only the direction changed and the noise manipulated.  Measures: Brain imagery, fMRI was used for imagery. Images were then pre-processed.  FMRI was also used for identifying regions of interest. | No significant difference found between both groups. Findings suggest that coherent motion perception may not be as impaired as first thought, or as previous studies have shown. FMRI showed no significant difference in brain structure in the coherent motion test. However, TD group showed more activity along the cortex in the biological motion test than the ASD group. |
| *McCleery, Allman, Carver & Dobkins (2007)* | 13 (in the matched analysis) (5 female, 8 male) 6-month old high risk infants (due to older siblings having ASD) and 5 additional (non-matched analysis) infants were used for this study. The older sibling’s diagnoses were confirmed using the ADOS and the ADI-R. The mean age for which the older siblings were clinically diagnosed from was 2.4years.  The mean age of high risk infants on first day of testing was 182.2 days and 182.1 days for the low risk infants. | YES |  |  | Visual perception was the main focal point of this study, focusing on measuring sensitivity of the Magnocellular (M) pathway and the Parvocellular (P) visual pathway, employing two visual stimuli to selectively stimulate the two. | Findings from the study show that ASD may be associated with abnormal processing of luminance contrast, which is mediated by the Magnocellular pathway, suggesting an abnormal Magnocellular pathway may be a marker for Autism. Detection of an abnormal M pathway may lead to being able to intervene earlier in atypical development. |
| *O’Riordan and Plaisted (2001)* | 15 ASC children, mean age 9:2 and mean matrices score 28.  15 neurotypical children, mean age 8:7 and mean matrices score 26.  Ratio of 12 male: 3 female.  ASC diagnosed by meeting DSM-IV criteria. | YES |  |  | Experiment 1: Comprised of 3 search tasks. In task 1, the participants had to search for a target defined by colour and orientation. In tasks 2&3, participants were asked to find a target comprised of 3 features; colour, size and orientation. All three tasks varied due to degree of target distractor similarity. Response times were recorded.  Experiment 2: the experiment utilised 4 search tasks, in which similarity was manipulated. Task 1 was a baseline task, asking participants to distinguish a red X hidden amongst green X and red C distractors. The other 3 tasks were modified versions of the first tasks, with different letters and colours being utilized. | Experiment 1: Children with Autism were found to be significantly faster on the search task. The findings show that the autism group had significantly faster response times than the control group. Findings suggest a superior nonverbal ability in those with autism.  Experiment 2: Results from this experiment show that those with autism did not significantly outperform the control group, but were less affected by the target distractor than the control group. Similar to experiment 1, findings suggest a superior nonverbal ability in those with autism. |
| *O’Riordan, Plaisted, Driver and Baron-Cohen (2001)* | Experiment 1: 12 children with Autism, with ages ranging from 6:11 to 9:6, Matrices Score Mean 26.  12 developmentally normal children, with ages ranging from 6:5 to 10:9, Matrices Score Mean 26.  Experiment 2: 12 children with Autism, with ages ranging from 7:1 to 9:7, Matrices Score Mean 28.  12 developmentally normal children, with ages ranging from 6:5 to 10:5, Matrices Score Mean 26.  ASC diagnosis confirmed by ADI-R. | YES |  |  | Experiment 1: The experiment consisted of 2 different search tasks, one search task focused on finding a feature target unique in terms of form and the other search task focused on finding a target defined by a combination of colour and form.  Experiment 2: This experiment again involved 2 search tasks, focusing on stimuli that were comprised of 2 possible items. In one task, one of the two items was a target and the other a distractor and in the other task, this was reversed. In the one task, the target was a slightly tilted line amongst vertical line distractors, and in the other task the vertical line was the target, and the tilted lines the distractors. | Experiment 1: The findings from the study show children with autism performed better on the second search task than their NT peers.  Experiment 2: The autism group performed better than the control group as response times overall were quicker and were significantly faster in the vertical line vs titled lines condition that counterparts. |
| *Pellicano et al (2005)* | 20 ASD children, mean age 9:6 and mean matrices score 40.  20 typically developing controls, mean age 9:9 and mean matrices score 41.  Diagnosis confirmed by the DSM-IV and the PDD. | YES |  |  | Participants were required to complete a series of 3 tests; the Global Dot Motion Task (indicating motion by pressing up or down on a button box), the Flicker Contrast Sensitivity Task (identifying which interval the stimulus occurred in) and the Children’s Embedded Figures Task (name complex picture, and locate hidden figure as quick as possible). | Global Dot Task: ASD group were outperformed by the Typically developed group.  Flicker Task: No difference in performance across both groups, suggesting no impairment in the corsal sensitivity of the dorsal pathway in ASD.  Children’s Embedded Figure Task: ASD group were significantly faster at finding the hidden figure than the TD control group. |
| *Price, Edgell, Kerns (2012)* | 14 males with Asperger Syndrome, mean age 14.14, FSIQ >70  16 typically developing, mean age 14.08, FSIQ >70  AS diagnosis confirmed by GADS, ASDI, and the ASSQ. | YES | YES |  | The Dean–Woodcock neuropsychological battery (Dean & Woodcock, 2003) was used to measure gross and fine motor skills.  Visual motion perception was assessed using a random dot kinematogram where participants viewed two adjacent patches of moving dots and had to identify which patch was oscillating.  Participants also completed a static form perception task and biological motion perception task  For postural stability participants stood on an AMTI AccuSway force plate, which depicted both a static and dynamic scene whilst measuring shifts in the centre of pressure. | This study proposed that participants on the autism spectrum are compromised in their ability to visually sense human motion and moving environments, but not necessarily motion in general |
| *Remington, Swettenham & Lavie (2012)* | 16 adults with ASC (11 males, 3 females), mean age 24.1, mean FSIQ 119  16 typical adults (6 males, 8 females), mean age 25.9, mean FSIQ 121  ASC diagnosis confirmed by ADOS. | YES |  |  | Microsoft Visual Basic (version 6) was used to create a computer based signal-detection paradigm | Showed that ASC adults were found to have enhanced perceptual capacity in visual detection. |
| *Ronconi et al., (2012)* | Eleven males with ASC, mean age 13.2, mean FSIQ 105.9  Twelve children, (9 males, 3 females) without ASC, mean age 13.4, mean FSIQ not given, subtests given.  ASC diagnosis confirmed by ADOS. | YES |  |  | Response latencies to a visual target onset displayed at three eccentricities from the fixation – using E-Prime 1.1 Software | Demonstrated an imbalance between sensory feedback and feedforward programs in children with ASC, when specific zoom-out visual attention was impaired. This abnormal attentional focusing is suggested to contribute to the atypical visual perception associated with ASC which, in turn, could have consequences in their social-communicative development. |
| *Smith and Milne (2009)* | 15 adolescents with ASD mean age 14y 0m, matrices score 31.9.  15 typically developing (TD) controls, with a mean age of 14y 6m, matrices score 40.5.  ASC diagnosis confirmed using the Childhood Autism Rating Scale (CARS). | YES |  |  | Participants were told to watch a film that contained mistakes, and were asked to look out for these mistakes (continuity errors). After each clip, participants were asked if they noticed any mistakes, and if so to describe them. Participants were also asked two questions, which encouraged them to remained focus. | Findings show that the ASC group had superior ability in detecting errors in the film clips shown to them, in comparison with the TD control group. |
| *Spencer et al. (2000)* | 23 children with Autism  50 controls matched for verbal mental age. | YES |  |  | Motion coherence thresholds were tested using a random array of moving dots on an LCD screen.  The task was to identify a target strip, shown on either the left or right hand side of screen, in which the dots oscillated in opposite phase to those in the surrounding region. | Motion coherence thresholds were much higher in participants with Autism than matched controls, with only a small difference in form coherence.  The disparity between motion and form coherence suggests that children with autism show a particular deficit on tasks that predominantly require processing through the dorsal stream. |
| *Stevenson et al., (2104)* | 31 children with ASD 31 TD children age and IQ matched | YES |  |  | Participants were given simple visual (i.e., flash) and auditory (i.e., beep) stimuli varying in number. For example, a single flash was presented with 2–4 beeps. In TD children this procedure should elicit the perception of multiple flashes, suggesting a perceptual fusion across the auditory visual modalities. | This study demonstrated that children with ASD were significantly less likely to perceive the illusion of multiple flashes that the TD controls. This suggests that multisensory integration and cross-modal binding could be impaired in some children with ASD. |
| *Vandenbroucke et al., (2009)* | 13 subjects (11 males, 2 females) with ASD, mean age 20.8, mean FSIQ 120.5.  31 control subjects (28 males, 3 females), mean age 21.6, mean FSIQ 117.3.  ASC diagnosis confirmed by ADOS and ADI-R. | YES |  |  | A new texture discrimination task was administered, where surface segregation was varied independently from orientation boundaries, | Proposed that the incorrect perceptual interpretation in tasks was due to relatively high levels of visual feedback. |
| **Sensory Processing - Proprioception** | | | | | | |
| *Blanche et al., (2012)* | 32 children with ASC, mean age 6.3 (3-10)  26 children with developmental disabilities excluding ASC, mean age 6.8 (3-10)  28 typically developing control children, mean age 6.7 (4-10)  ASC diagnosed independently by clinician | YES |  |  | Comprehensive Observations of Proprioception | Evidence suggested that children with ASC present with distinct patterns of proprioceptive processing difficulties, particularly related to activities requiring sensory feedback such as running, falling, crashing, tiptoeing and pushing objects. |
| **Sensory Processing - Auditory** | | | | | | |
| *Alcantara et al. (2004)* | 11 HFA participants, mean age 20.9 and a mean FSIQ of 105.6.  9 control participants, mean age 19.3 and a mean FSIQ of 106.  ASC diagnosis confirmed using ICD-10. | YES |  |  | Participants were required to correctly identify key words in a sentence with a distracting background noise. The level of the speech was decreased once participants correctly identifies 2 out of 3 key words, and level of the speech was increased for scores fewer than 2. | Overall findings show that HFA participants performed significantly worse that controls. HFA participants performed the worst in conditions that contained temporal or spectral dips, but not significantly different on ones that contained only spectral dips or no dips at all. |
| *Bonnel et al. (2003)* | 12 participants with HFA (11 male, 1 female), mean age 17.91 and a mean FSIQ score of 108.08.  12 normally developing adolescents (12 male), mean age 16.58 and a mean FSIQ score of 107.75.  The diagnosis of HFA was confirmed using the ADI-R and ADOS-G. | YES |  |  | An Audiometric Hearing test utilized for this study in 2 experiments. Participants were required to press a button upon hearing a tone. Each ear was tested individually.  Experiment 1 (Pitch Discrimination): Stimuli were presented at 100-msec long bursts, presented in pairs. The first tone was one of 4 values (500, 750, 1000 or 1500 Hz), followed by an identical tone or a higher pitched frequency.  Experiment 2 (Pitch Categorization): Tone presented to participants, which they had to categorize as a high or low tone. | Findings show that the normally developing group performed worse on the categorization task than the discrimination task. The ASD group, however, performed both tasks very similarly.  As a whole, ASD participants performed significantly better than the ND group. |
| *Heaton, Hudry, Ludlow & Hill (2008)* | 14 males with ASC, mean age 126 months, mean VMA 65.  14 children (13 male, 1 female) with either moderate learning difficulties or controls, mean age 126 months, mean VMA 64.  Diagnosis of ASC confirmed by school selection and criteria for ICD-10. | YES |  |  | Speech stimuli were presented as words, nonwords and nonspeech with varied pitch differences. Individuals were asked to identify whether there were any pitch differences between paired stimuli. | Results indicate that sensitivity to pitch cues is significantly heightened in ASC |
| *Jarvinen et al (2008)* | Experiment 1:  20 participants with Autism (17 male, 3 female), mean age 12.55, mean matrices score 87.  20 control participants (16 male, 4 female), mean age 12.03, mean matrices score 84.    Experiment 2:  20 male children with Autism, mean age 12.86, mean matrices score 85.  20 control participants (16 male, 4 female), mean age 12.86, mean matrices score 79.  Diagnosis of Autism confirmed using DSM-IV criteria. | YES |  |  | Experiment 1: Participants were required to match the sound heard to the physical stimuli that they felt had the best fit. To be able to continue, participants had to correctly judge 2 out of 4 stimuli. After, the participants were then told to listen to the same sentence again and listen for the meaning of what was said. Participants were also required to match melodies to the visual display.  Experiment 2: Similar to Experiment 1, however participants were told they were going to short sentences along with some voices/sound strings, which were going to be said in a certain way. | Experiment 1: Findings from the experiment show that neither group showed impaired semantic abilities, with similar levels of performance across conditions were shown.  Experiment 2: Findings suggest that participants with autism show superior processing of perceptual components of speech. However, linguistic comprehension performance was slightly worse in the autism group than the controls. |
| *Madsen, Bilenberg, Cantio & Oranje (2014)* | 35 children with ASC and 40 typical controls matched on age, IQ, gender and socio-economic status, aged 8-12 years old. ASC diagnosis confirmed by ADOS and ADI-R. | YES |  |  | *Copenhagen Psychophysiological*  *Test-Battery;* Participants startle reflex in response to loud auditory stimuli (105dB) was recorded by eye blink (EMG of the musculus orbicularis oculi), randomly interspersed with mild pre-pulse auditory stimuli (76 and 85 dB). | Participants with ASC showed a higher startle reflex than matched controls. Suggests problems with sensory hypersensitivity. |
| *O’Riordan and Passetti (2006)* | Experiment 1 & 2:  12 children with HFA, mean age 8:7, mean matrices score 26.3.  12 typically developing children, mean age 8:7, mean matrices score 26.5.  Experiment 3:  13 children with HFA, mean age 10:0, mean matrices score 34.5.  13 typically developing children, mean age 10:0, mean matrices score 35.9.  Autism diagnosis confirmed using ADI-R | YES |  |  | Experiment 1:  Participants heard sequences of two alternating tones. One tone stayed the same; the other changed in frequency and became lower until the two tones were identical. Participants were asked to indicate by pressing the button when they thought the two tones were identical.  Experiment 2:  Participants were asked to discriminate tactile differences between different types of sandpaper by indicating which sandpaper in a trial of pairs was rougher. This was then measured using the signal detection theory.  Experiment 3:  Different synthetic fibres were placed on the participants forearm until it began to bend. Each participant was required to state when they could feel the pressure of the materials on their arm. | Experiment 1:  Individuals with ASC indicated that the ones were identical significantly later than controls.  Experiment 2:  Both groups performed comparably overall.  Experiment 3:  No differences were apparent between groups for the detection of various degrees of tactile pressure.  These results demonstrate superior auditory but comparable tactile discrimination between ASC children and controls. |
| *Takahashi et al., (2014)* | 16 children and adolescents with ASC (9 male) and 30 age, gender and IQ matched typical control children (15 male), aged 6-17 years. ASC diagnosis confirmed with ADOS and ADI-R. IQ>70. | YES |  |  | SRS, acoustic startle response stimuli while eye blink was measured. | Children with ASC showed larger startle responses (higher peak amplitude of orbicularis oculi muscle), only in response to weak acoustic stimuli (<85dB). Startle reflex was also significantly prolonged in the ASC group. These behaviours were correlated with autistic traits. |
| *Teder-Sälejärvi et al. (2005)* | 7 autistic males, mean age 33.3 and a mean FSIQ 86.9.  7 control participants, mean age 33.4.  Autism diagnosis was confirmed using the ADI-R, ADOS-G and the CARS. | YES |  |  | EEG was used in this study on participants. Participants were required to press a button to the noise bursts at the location of the sound whilst being distracting by other sounds. | Results show that the control groups were more accurate in detecting the target source of the sound bursts whilst also having a faster reaction time in comparison with the autistic group.  Findings from the study suggest that the autistic group have difficulty in focusing on specific sources, implying a dispersed spatial awareness. |
| **Sensory Processing - Correlation Between Social Communication** | | | | | | |
| *Gepner and Mestre (2002)* | 3 children with Autism (1 male, 2 females) aged 7, 9 & 11  3 children with Asperger’s syndrome (3 males) aged 5, 7 & 9, FSIQ 85-115  ASC diagnosed independently by clinician  9 control children (5 boys, 4 girls).  ASC diagnosed independently by clinician | YES |  |  | Visual stimuli were generated using an image processing system | Confirmation of the existence of a visuopostural detuning in autistic children suggesting a correlation between visuopostural tuning and severity of motor signs in children with autistic spectrum disorders. |
| *Kern et al., 2007* | 104 participants with ASC, 79 male, aged 3-56 years old. | YES |  |  | The Sensory profile and Childhood Autism Rating Scale (CARS) were completed by a relevant professional familiar with the person. | Difficulties apparent across different sensory domains, which were highly correlated, suggesting global sensory dysfunction in ASC. Autism symptom severity only correlated with sensory problems in children 3-12 years. |
| *Lane, Young, Baker & Angley 2010* | 54 children with ASC, 47 male, aged 33-115 months. ASC diagnosis confirmed with ADI-R. | YES |  |  | The Short Sensory Profile (SSP) was completed by parents. The Vineland Adaptive Behavior Scales (VABS) data was extracted from patient records. | Children with ASC have significant differences in sensory processing. At least three distinct Sensory Processing subtypes of children with ASC; taste and smell sensitivity and movement related sensory behavior. Sensory processing subtypes were related to communication competence and maladaptive behaviours. |
| *Matsushima and Kato (2013)* | 42 children with ASC (36 male), and 42 age-matched control children (32 male), aged 48-72 months. | YES |  |  | The Social Responsiveness Scale (SRS) and the Japanese Sensory Inventory-Revised (JSI-R) completed by parents. | Difficulties in sensory processing in ASC with the exception of heightened olfactory sensitivity. Sensory Processing Disorder symptoms were significantly associated with social interaction difficulties in children with ASC. |
| *Tavassoli et al., (2013)* | 221 adults with ASC (206 males, mean age 38.7), and 181 age and IQ matched Adults without ASC (52 males, mean age 37.1). | YES |  |  | Autism Spectrum Quotient, Raven Matrices and the Sensory Processing Scale online. | Adults with ASC self-report significantly higher sensory over-responsivity than matched controls. Self-reported sensory over-responsivity and autistic traits were positively correlated in both groups. |
| *Tavassoli et al., (2014)* | 196 adults with ASC (100 males, mean age 39.7), and 163 age and IQ matched controls (49 males, mean age 36.8). | YES |  |  | Sensory Perception Quotient, the Sensory Over-Responsivity Inventory, the Autism-Spectrum Quotient (AQ) online and Raven Matrices online. | Adults with ASC self-report high sensory over-responsivity compared to controls across all domains except olfactory. Sensory sensitivity correlated with self-reported autistic traits in both groups. |
| **Sensory Processing - Synaesthesia** | | | | | | |
| *Baron-Cohen et al., (2013)* | 164 adults with ASC (mean age 39, 54% male), and 97 typical controls (mean age 41, 26% male). | YES |  |  | The Synaesthesia Questionnaire (self-report measure of synaesthesia symptoms), The Test of Genuineness-Revised (measures consistency of association between colours and particular sounds or letters), and the Autism Spectrum Quotient (AQ) online. | Found a significant increase in self-reported synaesthesia in ASC (18.9%) compared to controls (7.2%). This suggests that the two conditions may share some common underlying mechanisms, such as hyper-neural connectivity. |
| **Sensorimotor Integration - Difficulties** | | | | | | |
| *Gowen and Miall (2005)* | 12 participants with ASD (8 males, 4 females), mean age 27.4, mean FSIQ 104  12 typical controls (8 males, 4 females), mean age 28.2, mean FSIQ 112  ASC diagnosed independently by clinician and mean AQS 33.3 |  | . | YES | Tests examining visually guided movement (rapid pointing), speeded complex movement (finger tapping, rapid hand turning), muscle tone (catching dropped weight), prediction, coordination and timing (balance, grip force and interval timing)  A Polhemus electromagnetic motion tracking system, with an accuracy of 0.8 mm was used to measure the position of the finger, hand or body in x, y and z coordinates. | Evidence found of impaired motor control in ASC subjects. Differences were not found on all motor tests, but appeared most pronounced only on those tasks where accuracy depends upon incoming sensory signals such as in pointing, balancing and timing |
| *Iwanaga, Kawasaki & Tsuchida (2000)* | 10 children with Asperger Syndrome (7 males, 3 females, ages 58 to 74 months), mean FSIQ 93.3  15 children with autism (10 males, 5 females, 61 to 74 months) mean FSIQ 91.7 ASC diagnosed independently by psychologist and paediatrician. |  |  | YES | The Japanese version of the  Miller Assessment for Preschoolers (JMAP) (Tsuchida, Sato, Yamada, & Matsushita, 1989). | Results indicated that sensory-motor dysfunction might be more common in children with Asperger Syndrome than Higher Functioning Autism in early childhood. |
| *Salowitz et al., (2012)* | 12 children with ASC (10 male, 12-16 years old), and 14 IQ matched typical controls (11 male, 12-14 years old). FSIQ 85-115. ASC diagnosis confirmed by ADOS. |  |  | YES | Participants imitated gestures, and traced shapes with their hand in full view, or only in view of a mirror image. | ASC group could trace shapes with their hand in full view as controls, but had significant difficulties imitating others actions, or when tracing shapes with feedback from a mirror image. These difficulties were correlated, suggesting that difficulties with in visuospatial processing and motor coordination may be related. |
| **Sensorimotor Integration - Differences in Sensitivity to Visual and Proprioceptive Cues** | | | | | | |
| *Dowd, McGinley, Taffe & Rinehart (2012)* | 11 children with ASC (8 males, 3 females), mean age 6.2, mean FSIQ 88.2  12 typically developing children (9 males, 3 females), mean age 6.6, mean FSIQ 102.5  All diagnoses confirmed by first author using DSM-IV-TR criteria (American Psychiatric Association, 2000) | YES | YES |  | Movement kinematics were recorded with a computerized touch screen task using custom made software programmed  in C# using Microsoft Visual Studio. | In contrast to typically developing children, the presence of a visual distractor in the movement task did not appear to impact on early movement planning or execution in children with autism, suggesting that this group were not considering all available environmental cues to modulate movement. |
| *Glazebrook, Gonzalez, Hansen & Elliott (2009)* | 13 young adults with ASC (2 female), mean age 23.4, 3 left handed males  15 young adults without ASC (2 female), mean age 23.4, 3 left handed males  Verbal Age ability ranged between 6-33.7, and PIQ 65-107  ASC diagnosed independently by clinician a qualified health professional. |  |  | YES | Participants performed eye movements and/or manual reaching movements, either with or without vision using a custom made board attached to software designed with E-prime (version 1.1a) which synchronized presentation of the targets with the initiation of the Optotrak and eye tracker.  Eye tracker (Applied Sciences Laboratory (ASL) H6) that recorded eye movements at 60 Hz. | Individuals with autism used vision and proprioception. However, they took considerably more time to perform movements that required greater visual-proprioceptive integration than the participants without ASC. |
| *Nystrom et al., (2015)* | 29 high-risk infants with an ASC sibling (13 male), and 15 typical controls (8 male), aged 10 months, matched on CA and MA. | YES | YES |  | Eye tracking methods measured pupil dilation in response to flashing lights. | Infants at risk for developing ASC showed significantly higher pupillary dilation in response to light than typical controls. |
| *Paton, Hohwy, & Enticott (2012)* | 17 participants with high functioning ASC (14 male, 3 female), mean age 32, 17 typical controls (12 male, 5 female) mean age 27, FSIQ>70.  ASC diagnosis confirmed independently and during prior research |  |  | YES | *Rubber hand illusion (RHI);* participants experienced synchronous vs. asynchronous touch on their hand, a rubber hand or box, while their arm was occluded. | The ASC group did experience the RHI, but had subtle differences in sensitivity to visuotactile and proprioceptive cues. |
| *Siaperas, et al., (2011)* | 50 males with ASC, aged 7-14 years  50 males without ASC.  ASC diagnosis confirmed by ADI-R. | YES | YES |  | The Movement Assessment Battery for Children–2 (MABC-2; Henderson et al. 2007)  The Sensory Integration Praxis Test (SIPT; Ayres 1989) | Showed that overall, children with ASC showed significant impairment of movement performance as well as proprioceptive and vestibular processing. |
| *Wilkes et al., (2015)* | 16 children with ASC and 24 typical controls aged 6-12 years, FSIQ>70. ASC diagnosis confirmed with ADOS and SCQ. |  |  | YES | Children looked at a moving light while eye movements were measured. | Children with ASC showed no difficulties in smooth visual pursuit, but a delayed latency in initiating saccades. |
| **Sensorimotor Integration - Cerebellum Function in ASC** | | | | | | |
| *Marko et al., (2015)* | 20 children with ASC (18 male) and 20 typical controls (16 male) aged 8-12, and matched on age, gender, PIQ and handedness.  ASC diagnosis confirmed with ADOS and ADI-R. |  |  | YES | Participants moved a robotic manipulandum through a designated target. Proprioceptive and visual cues randomly perturbed the participants’ movement. MRI mapping of cerebellum volume. | Children with ASC outperformed typical controls when learning from proprioceptive errors, but were significantly less accurate when learning from visual errors. Relationship between cerebellum volume involved in sensorimotor processing, and learning from both proprioceptive and visual errors. |
| *Mosconi et al., (2013)* | 56 participants with ASC (50 male) and 53 typical controls (46 male), aged 8-54 years, matched on age and gender. ASC diagnosis confirmed with ADOS and ADI-R. FSIQ above average. |  |  | YES | *Saccade adaptation task*; participants move fixation from a central target to a peripheral target in the horizontal plane, in adaptation trials the target location is shifted directly after the start of the saccade.  *Manual motor test;* participants place pegs in a board as quickly as possible. | ASC group had significant difficulties in rapidly adjusting large errors in fixation on a target in adaptation trials, and control over the consistency of eye movements. This suggests atypicalities in cerebellum responsible for motor learning. |
| *Mostofsky et al., (2000)* | 11 ASC (6 male) and 17 age and IQ matched typical controls (6 male), aged 6-17 years, FSIQ 80 – 130. ASC diagnosis confirmed with ADI-R and ADOS. |  | YES |  | *Judgment of Timing*; participants judged whether the gap between two previous tones was longer or shorter than the current gap between tones.  *Procedural Learning*; participants press a corresponding key to indicate which of four circles light up across a computer screen. Acquisition of procedural knowledge is indicated by increasingly faster response times. | The ASC group showed no significant difficulties in judgment of timing, but significant difficulties in acquisition of procedural knowledge. This implicates dysfunction in the cerebellum in ASC. |
| *Schmitt, Cook, Sweeney & Mosconi (2014)* | 65 participants with ASC and 43 typical controls, aged 6-44 years old, matched on age, non-verbal IQ and gender. ASC diagnosis confirmed with ADOS and ADI-R. FSIQ >80. |  |  | YES | Saccades in blocked gap and overlap trials; participants fixate peripheral targets from a central fixation point. In gap trials, the central fixation disappears 200ms before onset of the peripheral target. In overlap trials, the central fixation overlaps for 200ms with onset of the peripheral target. | ASC group showed reduced accuracy of saccades to the peripheral target, increased variability of saccades across trials, lower peak velocity and duration of saccade, increased time to reach peak velocity in addition to difficulties in decelerating saccades. This suggests unique atypicalities in cerebellum and brain stem in ASC. |
| **Sensorimotor Integration - Feedback / Feedforward Imbalance** | | | | | | |
| *Gowen, Stanley and Miall (2008)* | 12 ASD individual (6 males, 6 females) (3 excluded in trial), mean age 33.9, mean FSIQ 117.6,  12 typical controls (6 males, 6 females), mean age 32.0, mean FSIQ 115.7.  ASC diagnosed independently by clinician |  | . | YES | Movement recording and signal processing was measured using the following:  A visual stimulus was created, a 1-cm diameter white dot presented on a black background.  A metronome for self-pacing movements was a sequence of tones presented at 1Hz over headphones. Fingertip position was recorded using the Optotrak 3020 active marker system (Northern Digital Instruments, Inc.). | Results were interpreted as evidence that the ASC participant group either relied to a greater extent on the goal-directed feedforward imitation pathway, supporting claims that they have a specific deficit of the non-goal-directed imitation pathway, or exhibit reduced visuomotor integration |
| *Izawa et al., (2012)* | 23 children with ASD (20 males, 3 females), mean age 10.4, PRI >80  17 children with ADHD (14 males, 3 females, mean age 10.8, PRI >80 (with the exception of one child 79)  20 TD children (16 males, 4 females) mean age 10.9, PRI >80  ASC diagnosis confirmed by ADI-R, ADOS and SRS. |  | YES | YES | Imitation was assessed as a part of a praxis examination adapted from the Florida Apraxia Battery modified for children (Mostofsky et al. 2006),  Basic motor skills assessed by the Revised Physical and Neurological Examination  of Subtle Signs (PANESS) (Denckla, 1985).  Reach adaptation Task paradigm using a robotic arm from Haswell et al. (2009). | Demonstrated that the motor memory in children with ASC relies more heavily on proprioceptive (sensory) feedback as opposed to a predetermined feedforward (motor) program |
| *Schmitz, Martineau, Barthélémy & Assaiante (2003)* | Eight right-handed children with autism (6 males, 2 females), mean age 7.9, FSIQ >70.  Sixteen healthy right-handed children (7 males, 9 females), mean age 6.0.  ASC diagnosis confirmed by trained child psychiatrist and CARS. |  |  | YES | Participants completed a bimanual load-lifting task, which involved lifting a load placed on the controlateral forearm and stabilising the postural forearm that supported the load.  Force and angular elbow displacement signals were recorded and digitised (sampling rate: 500 Hz) along with EMG signals for analysis | Proposed that the motor memory in children with ASC has been shown to rely more heavily on proprioceptive (sensory) feedback as opposed to the predefined feedforward program |
